# Development of a bispecific antibody targeting PD-L1 and TIGIT with optimal cytotoxicity

**DOI:** 10.1038/s41598-022-22975-7

**Published:** 2022-10-26

**Authors:** Zhenwei Zhong, Mengyao Zhang, Yanan Ning, Guanchao Mao, Xiaopei Li, Qi Deng, Xiaorui Chen, Dongliang Zuo, Xiangyu Zhao, Ermin Xie, Huajing Wang, Lina Guo, Bohua Li, Kai Xiao, Xiaowen He

**Affiliations:** 1 Department of R&D, Oricell Therapeutics, Origincell Industrial Park, No. 1227 Zhangheng Road, Pudong New District, Shanghai, 201203 China; 2 Graduate School of Shanghai University of Traditional Chinese Medicine, Shanghai University of Medicine and Health Science, Shanghai, 201203 China; 3 Department of Protective Medicine Against Chemical Agents, Faculty of Naval Medicine, Naval Medical University, Shanghai, 200433 China

**Keywords:** Biochemistry, Biotechnology, Cancer, Drug discovery, Immunology

## Abstract

Programmed death-ligand 1 (PD-L1) and T cell immunoreceptor with Ig and ITIM domains (TIGIT) are two potential targets for cancer immunotherapy, early clinical studies showed the combination therapy of anti-PD-L1 and anti-TIGIT had synergistic efficacy both in the terms of overall response rate (ORR) and overall survival (OS). It is rational to construct bispecific antibodies targeting PD-L1 and TIGIT, besides retaining the efficacy of the combination therapy, bispecific antibodies (BsAbs) can provide a new mechanism of action, such as bridging between tumor cells and T/NK cells. Here, we developed an IgG1-type bispecific antibody with optimal cytotoxicity. In this study, we thoroughly investigated 16 IgG-VHH formats with variable orientations and linker lengths, the results demonstrated that (G4S)2 linker not only properly separated two binding domains but also had the highest protein yield. Moreover, VHH-HC orientation perfectly maintained the binding and cytotoxicity activity of the variable domain of the heavy chain of heavy‐chain‐only antibody (VHH) and immunoglobulin G (IgG). Following treatment with BiPT-23, tumor growth was significantly suppressed in vivo, with more cytotoxic T lymphocytes (CTLs) and natural killer (NK) cells infiltration, and selective depletion of Regulatory T cells (Tregs). BiPT-23 represents novel immunotherapy engineered to prevent hyperprogression of cancer with PD-1 blockade, and preferentially killed PD-L1^+^ tumor cells, and TIGIT^+^ Tregs but maintained CD11b^+^F4/80^+^ immune cells within the tumor microenvironment (TME).

## Introduction

In the past two decades, bispecific antibodies provided massive opportunities for the treatment of clotting deficiency^1^ and cancer^2–4^. 272 clinical trials of bispecific antibodies proceeded from 1997 to 2020, BsAbs possessed hundreds of formats^5^ and about six mechanisms of action^6^, according to mechanism of action (MOA), different formats should be adopted. However, not all the formats are suitable for industrial manufacturing, IgG-like and BiTE have been proved to possess good developability and are patent-protected^7^. Since the discovery of VHH in 1993^8^, VHH got much attention from academic institutes and biotech companies. The approval of Caplacizumab by the U.S. Food and Drug Administration (FDA) in 2019 gave a boost for VHH^9^. Due to its small size (15 kDa), it is convenient to construct bispecific antibodies with VHH.

Among all clinical trials, 17.28% were dual checkpoint blockade^10^. Immune checkpoint inhibitors had the potential to redefine cancer immunotherapy. Recently, T cell immunoglobulin and ITIM domain (TIGIT) was found to be co-expressed with PD-1 on tumor-infiltrating lymphocytes (TILs)^11^, and the combination of PD-L1/PD-1 blockade with anti-TIGIT showed superior ORR and OS than PD-L1/PD-1 single agent in PD-L1 positive (tumor proportion score [TPS] ≥ 1%) cancer patients^12^. There are several bispecific antibodies under clinical development in China targeting PD-L1 and TIGIT^13,14^, however, none of them were optimized to induce antibody-dependent cellular cytotoxicity (ADCC) and antibody-dependent cellular phagocytosis (ADCP).

In tumors, PD-L1 is expressed on both tumor cells^15^ and immune cells^16^, and preclinical data revealed that functional Fc could enhance the anti-tumor activity of PD-L1 antibodies via the ADCC against the PD-L1 positive immune-suppressive myeloid cells^17^ and tumor cells^18^. Several PD-L1 antibodies approved or in clinical trials are IgG1 monoclonal antibodies with effector function^19^. Recently, researchers developed an anti-TIGIT monoclonal antibody (mAb), T4, which caused a significant reduction in the frequency of intratumoral T regulatory cells and activated additional antitumor CD8^+^ T cell responses^20^. Subsequent Fc engineering to enhance FcγR engagement capability further improved antitumor efficacy^20^. In addition, the interaction with FcγR on APCs could improve antigen-specific T cell responses for anti-TIGIT^21^. In summary, Fc-FcγR interaction for PD-L1 and TIGIT blockades not only helped NK to deplete tumor cells and Tregs but also strengthened the cross-talking between T cells and antigen-presenting cells (APCs).

In this study, we systematically investigated the relationship between BsAb’s cytotoxicity and IgG-VHH formats. First, we constructed 16 BsAbs based on IgG and VHH, and demonstrated that the connection of VHH with the light chain of IgG weakened the pair between HC and LC. Next, we confirmed that putting the VHH at the N terminus of the heavy chain maintained the cytotoxicity of parental VHH, but not at the C terminus. Last, in vivo antitumor activity showed that the top candidate could suppress the growth of MC38 colon carcinoma, reduced the frequency of intratumoral Treg cells, increased NK and T cells, and maintained CD11b^+^F4/80^+^ immune cells, including DCs and macrophages within the TME.

## Results

### Design of tetravalent bispecific antibodies targeting PD-L1 and TIGIT

**Figure 1:**
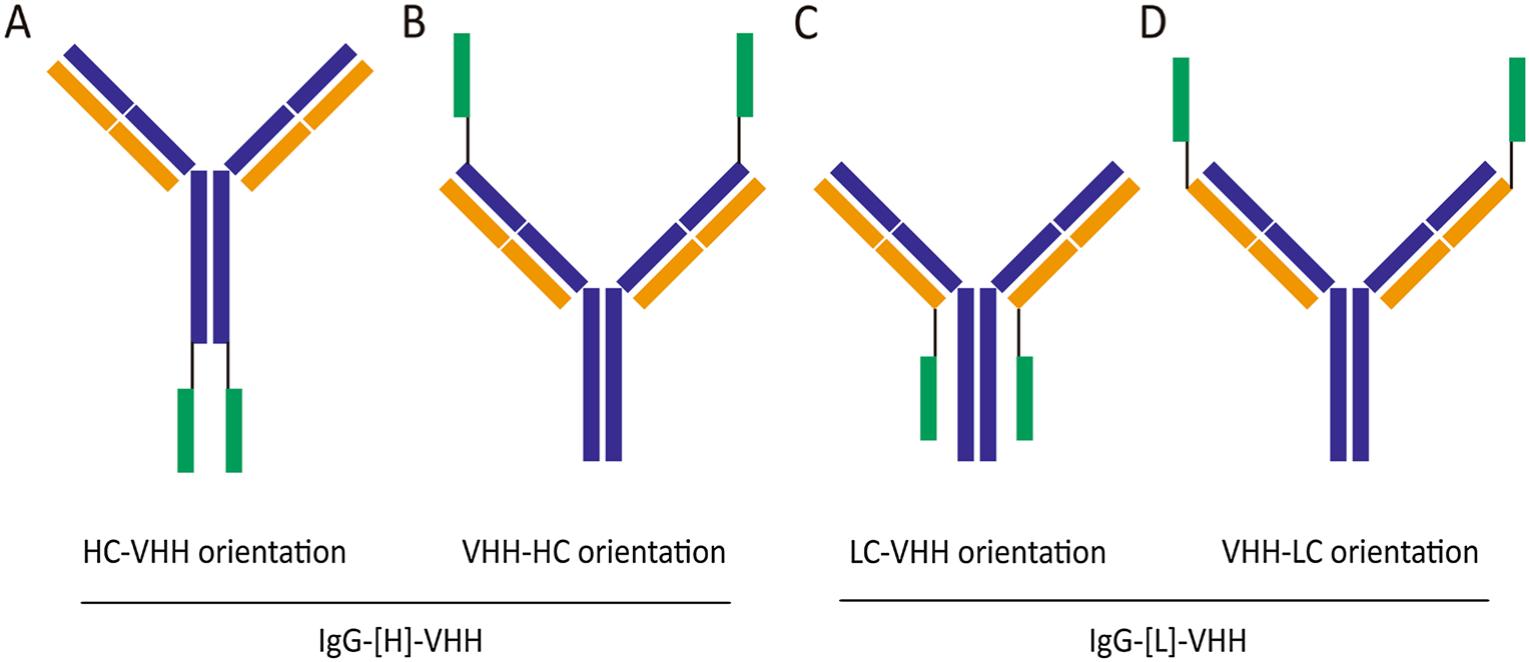
The design of bispecific antibodies. Green is the VHH against TIGIT, deep blue is the heavy chain of anti-PD-L1, brown is the light chain, and black is the linker: (GGGGS)n, n = 0–3.

YN035 (IgG, anti-PD-L1) and hTIGI7.6, hTIGI7.11E (VHH, anti-TIGIT) were developed by Oricell Therapeutics previously. hTIGI7.6 and hTIGI7.11E from the same progenitor sequence of hTIGI7 had only a few amino acid differences in CDR3. Based on YN035 and hTIGI7.6, we designed 16 bispecific antibodies (appended IgGs) with different formats and linker lengths (Fig. 1). Bivalency not only could improve conjugate formation, which may help TIGIT-expressing T/NK cells get close to PD-L1 tumor cells^22^, but also maintained the blocking activity of parental antibodies. The best option of the flexible and soluble linker was (G4S)n, which applied to connect domains that required a certain degree of movement^23^. The longest linker was (G4S)3 which was calculated to be about 5.7 nm^23^, shorter than the distance of immune synapse (13 nm)^24^. By easily adjusting the number of n, the linker allowed BsAbs for proper folding, achieving the highest yield and optimal biological activities. In our design, n ≥ 4 was avoided, on the one hand, (G4S)3 was long enough to separate two binding domains^25^; on the other hand, GSG is the motif for O-xylosylation and the total amount of xylosylation increases as the number of GSG motifs increases in the linker^26^.

### IgG-[H]-VHH bispecific antibodies were more stable than IgG-[L]-VHHs

**Figure 2:**
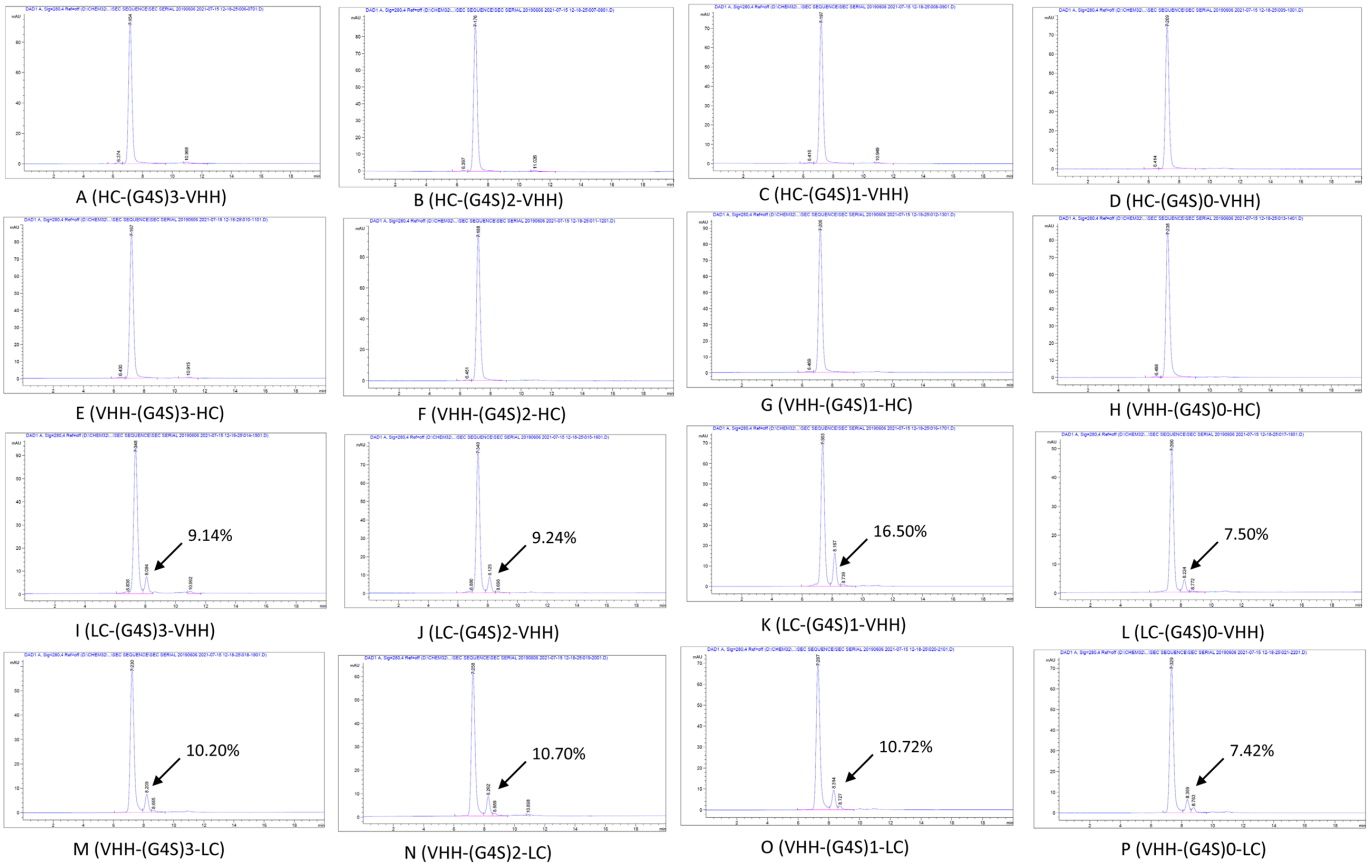
SEC of 16 bispecific antibodies at 1 mg/ml concentration. (**A**–**H**) IgG-[H]-VHH formats showed no aggregation and no peak of heavy chain only. (**I**–**P**) IgG-[L]-VHH formats had no aggregation peak, however, the connection of VHH to the light chain weakened the association of HC and LC, there was a small peak at elution time of 8.1–8.4 min.

All BsAbs have been expressed in EXPI293 cells (Thermo Fisher Scientific, A14527CN), after purification with protein A, the aggregation and dissociation of BsAbs were evaluated using analytical size exclusion chromatography (SEC) (Fig. 2). In this assay, BsAbs were theoretically calculated to be 180 kDa, including one 150 kDa IgG and two 15 kDa VHHs, the BsAbs’ monomer elution time was about 7.1–7.4 min. No significant aggregation was observed for all BsAbs. However, at the elution time of 8.1–8.4 min, IgG-[L]-VHH formats (Fig. 2I–P) had a small peak (7–17%) which may be the heavy chain of BsAbs, no light chain was observed in SEC assay, because the dissociation occurred at expression or purification steps, not at storage. The results of the data demonstrated IgG-[H]-VHH bispecific antibodies were more stable than IgG-[L]-VHHs, so A-H formats (Fig. 2) were chosen for further analysis and constructed bispecific antibodies BiPT-18 to BiPT-25 based on YN035 and hTIGI7.11E.

### VHH-HC orientation had better cytotoxicity than HC-VHH orientation

**Figure 3:**
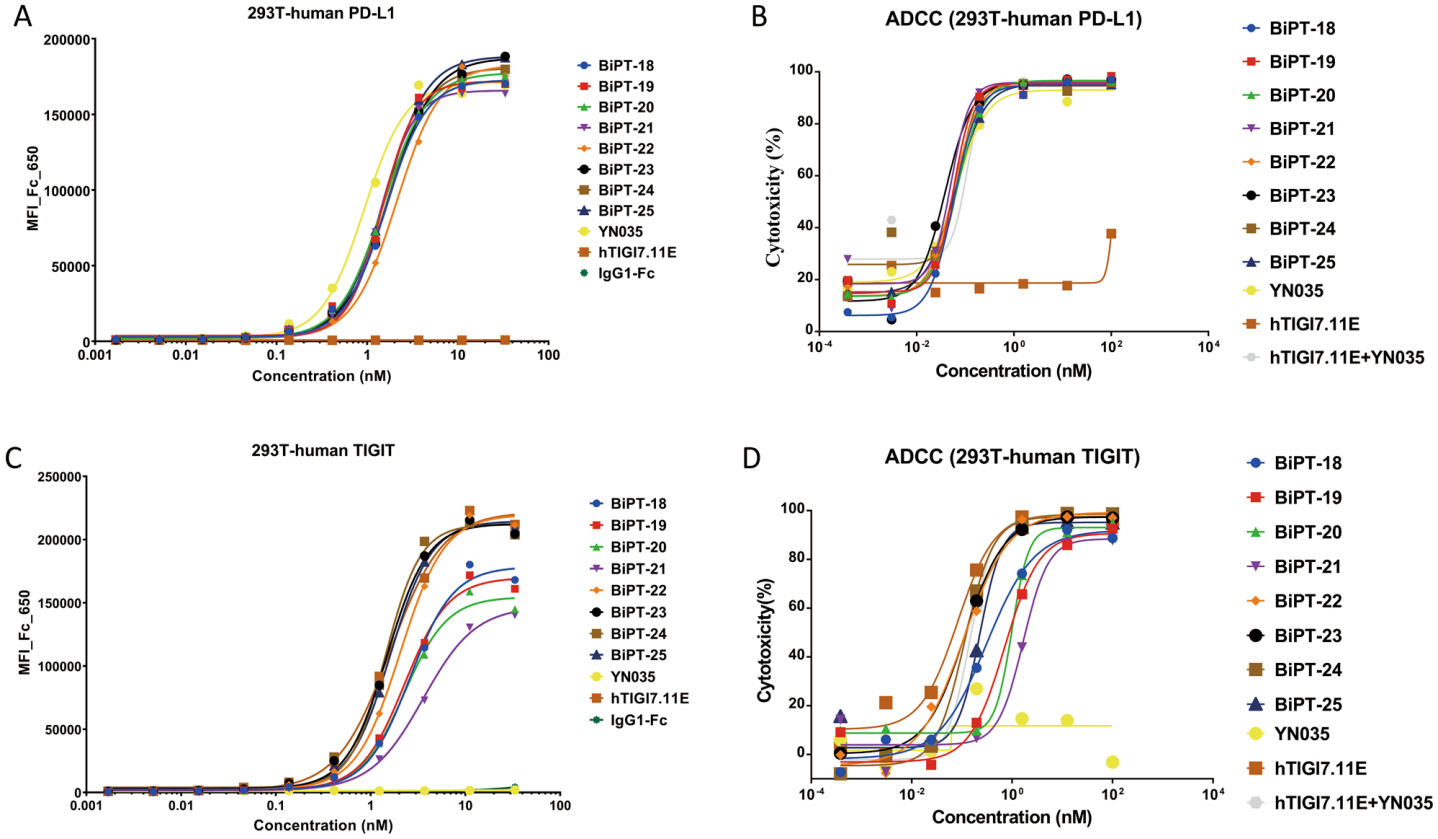
Binding and ADCC activities of BiPTs. (**A**) The binding ability to human PD-L1 is shown. (**B**) ADCC activity to PD-L1-overexpressing 293 T cell line is shown. (**C**) The binding ability to human TIGIT is shown. (**D**) ADCC activity to TIGIT-overexpressing 293 T cell line is shown.

The binding ability of BiPT-18–25 was analyzed by flow cytometry using PD-L1 overexpressing 293 T cell line, to investigate the influence of VHH to IgG. Here, all IgG-[H]-VHHs bound PD-L1^+^ 293 T cells at the same level, both in the terms of maximum and half-maximal effective concentration (EC50) (Fig. 3A, Table 1). Compared with parental antibody YN035, BiPT-18–25 showed a 1.5–2.0 times loss in EC50, however, we did not see affinity reduction in the ForteBio assay (data not shown). In addition, to evaluate the potential impact of the VHH to the CD16a binding region on BsAbs, we performed in vitro cytotoxicity assays, all bispecific antibodies, YN035, hTIGI7.11E, and the combination of parental antibodies were measured with PD-L1^+^ 293 T and human peripheral blood mononuclear cell (PBMC) (Fig. 3B). All groups except hTIGI7.11E had the same level of cytotoxicity in terms of maximum lysis and EC50. These results indicated that VHH fusion to the heavy chain would not interrupt the binding of Fc to CD16a. We next evaluated the binding ability and cytotoxicity of the VHH arm of BsAbs. First, flow cytometry using TIGIT overexpressing 293 T cell line was conducted, and the results showed that BiPT-22–25 (VHH-HC orientation) maintained the binding activity of hTIGI7.11E, BiPT-18–21 (HC-VHH orientation) showed a significant reduction in binding, and the binding loss intensified as the linker length became shorter (Fig. 3C, Table 1). There was a steric hindrance for antigen recognizing, when the VHH was fused to the C-terminus of the heavy chain. The same results could be observed in the cytotoxicity assay, BiPT-18–21 performed inferior in cell lysis, compared with BiPT-22–25 (Fig. 3D, Table 1). In summary, linker length is critical for VHH’s binding and cytotoxicity in HC-VHH orientation, but not in VHH-HC orientation. Considering the importance of cytotoxicity for TIGIT antibodies, BiPT-22, 23, 24, and 25 were better candidates for further development.

**Table 1:** Summary of bispecific antibodies.

ID	BiPT-ID	IgG	VHH	Fc	Format	Orientation	Linker	SEC of BsAbs	Stable	EC50/nM/Binding PD-L1	EC50/nM/Binding TIGIT	EC50/nM/ADCC PD-L1	EC50/nM/ADCC TIGIT	Yield/mg in 100 ml	Yield/mg in 300 ml
A	/	YN035	hTIGI7.6	IgG1-Fc	IgG-[H]-VHH	HC-VHH	(G4S)3	96.55%	Yes	/	/	/	/	/	/
B	/	YN035	hTIGI7.6	IgG1-Fc		HC-VHH	(G4S)2	97.78%	Yes	/	/	/	/	/	/
C	/	YN035	hTIGI7.6	IgG1-Fc		HC-VHH	(G4S)1	97.19%	Yes	/	/	/	/	/	/
D	/	YN035	hTIGI7.6	IgG1-Fc		HC-VHH	(G4S)0	99.33%	Yes	/	/	/	/	/	/
E	/	YN035	hTIGI7.6	IgG1-Fc		VHH-HC	(G4S)3	98.04%	Yes	/	/	/	/	/	/
F	/	YN035	hTIGI7.6	IgG1-Fc		VHH-HC	(G4S)2	98.97%	Yes	/	/	/	/	/	/
G	/	YN035	hTIGI7.6	IgG1-Fc		VHH-HC	(G4S)1	98.85%	Yes	/	/	/	/	/	/
H	/	YN035	hTIGI7.6	IgG1-Fc		VHH-HC	(G4S)0	99.23%	Yes	/	/	/	/	/	/
I	/	YN035	hTIGI7.6	IgG1-Fc	IgG-[L]-VHH	LC-VHH	(G4S)3	87.15%	No	/	/	/	/	/	/
J	/	YN035	hTIGI7.6	IgG1-Fc		LC-VHH	(G4S)2	87.78%	No	/	/	/	/	/	/
K	/	YN035	hTIGI7.6	IgG1-Fc		LC-VHH	(G4S)1	81.71%	No	/	/	/	/	/	/
L	/	YN035	hTIGI7.6	IgG1-Fc		LC-VHH	(G4S)0	91.25%	No	/	/	/	/	/	/
M	/	YN035	hTIGI7.6	IgG1-Fc		VHH-LC	(G4S)3	88.10%	No	/	/	/	/	/	/
N	/	YN035	hTIGI7.6	IgG1-Fc		VHH-LC	(G4S)2	86.67%	No	/	/	/	/	/	/
O	/	YN035	hTIGI7.6	IgG1-Fc		VHH-LC	(G4S)1	86.41%	No	/	/	/	/	/	/
P	/	YN035	hTIGI7.6	IgG1-Fc		VHH-LC	(G4S)0	89.24%	No	/	/	/	/	/	/
/	BiPT-18	YN035	hTIGI7.11E	IgG1-Fc	IgG-[H]-VHH	HC-VHH	(G4S)3	95.70%	Yes	1.599	2.588	0.053	0.357	2.835	80
/	BiPT-19	YN035	hTIGI7.11E	IgG1-Fc		HC-VHH	(G4S)2	98.27%	Yes	1.456	2.284	0.064	0.878	4.677	84.4
/	BiPT-20	YN035	hTIGI7.11E	IgG1-Fc		HC-VHH	(G4S)1	98.43%	Yes	1.495	2.258	0.072	0.816	2.769	75.14
/	BiPT-21	YN035	hTIGI7.11E	IgG1-Fc		HC-VHH	(G4S)0	97.49%	Yes	1.37	3.586	0.049	1.55	1.387	73.44
/	BiPT-22	YN035	hTIGI7.11E	IgG1-Fc		VHH-HC	(G4S)3	97.69%	Yes	2.114	2.056	0.073	0.106	1.991	76.16
/	BiPT-23	YN035	hTIGI7.11E	IgG1-Fc		VHH-HC	(G4S)2	97.97%	Yes	1.747	1.482	0.04	0.092	4.157	90.66
/	BiPT-24	YN035	hTIGI7.11E	IgG1-Fc		VHH-HC	(G4S)1	98.00%	Yes	1.639	1.43	0.062	0.143	3.744	90
/	BiPT-25	YN035	hTIGI7.11E	IgG1-Fc		VHH-HC	(G4S)0	97.01%	Yes	1.568	1.607	0.071	0.226	1.62	72

### (G4S)2 linker had the highest yield and BiPT-23 showed reasonable stability

**Figure 4:**
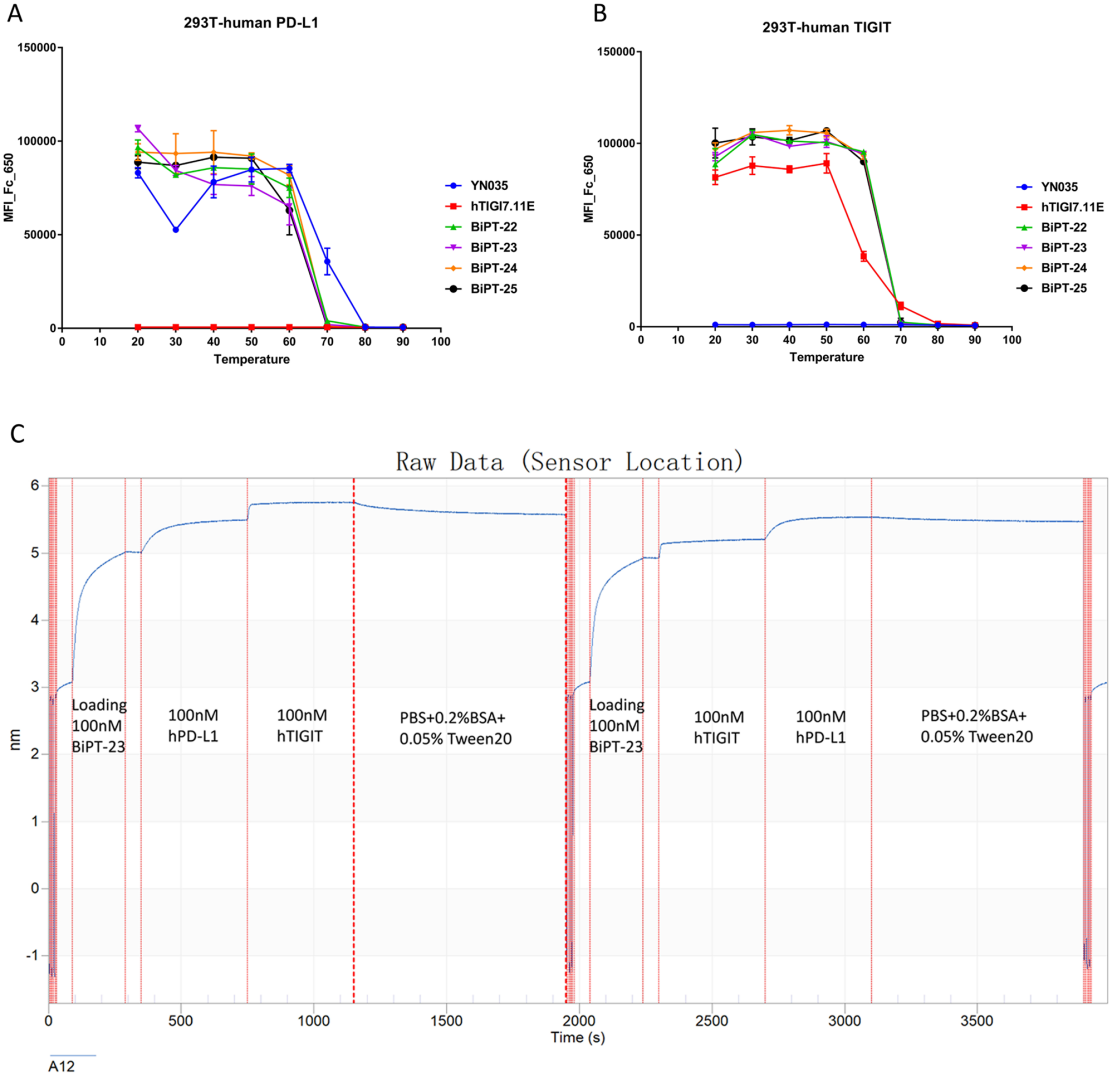
The stability assay of BiPT-22-25. (**A**) After the heating challenge at variable temperatures, 25 nM antibodies were incubated with PD-L1-overexpressing 293 T cell line to determine their binding ability. (**B**) After the heating challenge at variable temperatures, 25 nM antibodies were incubated with TIGIT-overexpressing 293 T cell line to determine their binding ability. (**C**) BiPT-23 could bind PD-L1 and TIGIT simultaneously in the ForteBio Octet assay.

Besides the biological activity of BsAbs, the expression yield and stability were also analyzed, BiPT-18–25 were expressed in 100 ml and 300 ml EXPI293 respectively, then purified under the same condition as materials and methods. The results showed that (G4S)2 linker had the highest yield both in HC-VHH and VHH-HC orientation (Table 1). The stability assay showed no difference among BiPT-22–25 (Fig. 4A,B), however, hTIGI7.11E lost half-binding ability to TIGIT at 60 °C, and BiPTs were unimpaired. The unexpected result revealed that IgG helped to stabilize the VHH in BsAbs. On the contrary, the denaturation of VHH at 70 °C disturbed the binding ability of BiPT-22–25 to PD-L1. Taking all the results into account, BiPT-23 was the best BsAb which could bind PD-L1 and TIGIT simultaneously (Fig. 4C). BiPT-23 was chosen for evaluating antitumor activity in the human TIGIT-KI C57BL/6 mouse model.

### BiPT-23 showed tumor-suppressing activity in human TIGIT-KI C57BL/6 mouse

**Figure 5:**
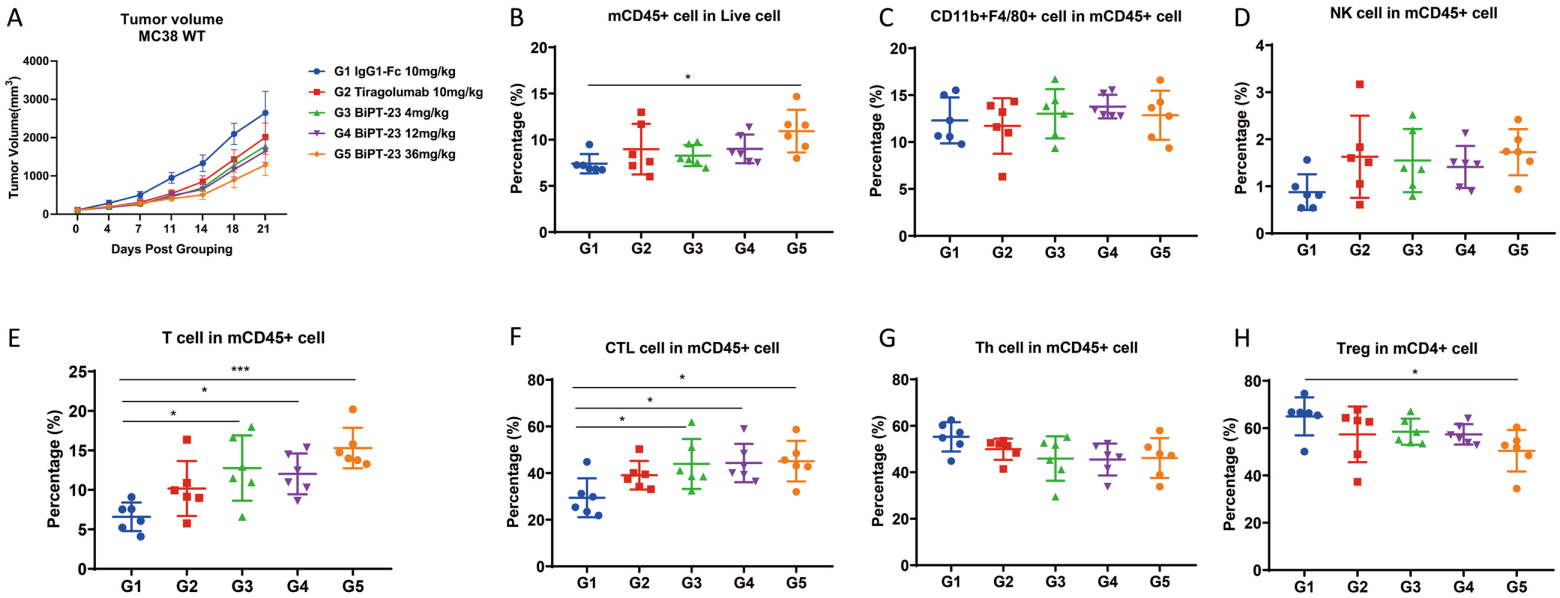
BiPT-23 inhibited tumor growth in vivo and preferentially increased NK and CTL cells, decreased Treg cells, and maintained CD11b^+^F4/80^+^ immune cells within the TME. (**A**) The antitumor activity of BiPT-23 with three doses. (**B**) Analysis of mCD45^+^ cells as a fraction of live cells within the TME is shown. (**C**) Analysis of mCD11b^+^ F4/80^+^ cells as a fraction of mCD45^+^ cells within the TME is shown. (**D**) Analysis of NK cells (CD3^-^NK1.1^+^) as a fraction of mCD45^+^ cells within the TME is shown. (**E**) Analysis of T cells (CD3^+^) as a fraction of mCD45^+^ cells within the TME is shown. (**F**) Analysis of CTL cells (CD4^-^CD8^+^) as a fraction of mCD45^+^ cells within the TME is shown. (**G**) Analysis of T helper (Th) cells (CD4^+^CD8^-^) as a fraction of mCD45^+^ cells within the TME is shown. (**H**) Analysis of Treg cells (CD4^+^FoxP3^+^) as a fraction of mCD4^+^ cells within the TME is shown. Data were shown as Mean ± SEM and analyzed using one-way ANOVA followed the Tukey test compared with each group. (*p < 0.05, ***p < 0.001).

Since YN035 had the cross-reactivation between human and mouse PD-L1, hTIGI7.11E only recognized human TIGIT, and human TIGIT could cross-recognize mouse poliovirus receptor (PVR)^27^. We used the TIGIT-humanized C57BL/6 mouse and MC38 colon carcinoma to evaluate in vivo efficacy and observed dose-dependent tumor regression. 36 mg/kg BiPT-23 showed 53.7% tumor growth inhibition (TGI_tv_), compared with the IgG1-Fc group (Fig. 5A). Flow cytometric analysis of immune cells within the TME showed that T cells significantly increased in three BiPT-23 groups (Fig. 5E). The increased T cells mostly were CD8 positive CTL (Fig. 5F), while CD4 positive Th cells showed a slight drop (Fig. 5G). As a previous report, TIGIT-overexpressing Treg cells significantly reduced duo to the ADCC activity of BiPT-23 (Fig. 5H). Moreover, we also detected the frequency of other PD-L1 positive immune cells, and BiPT-23 did not alter myeloid subset composition (Fig. 5C), which means BiPT-23 selectively depleted PD-L1^+^ tumor cells, other than PD-L1^+^ immune cells, consistent with another report^28^. NK cells also increased in Tiragolumab and BiPT-23 groups, although there was no statistical difference (Fig. 5D). In 2018, Zhigang Tian lab found that the blockade of TIGIT reversed the exhaustion of tumor-infiltrating NK cells, increased the frequency of tumor-infiltrating NK cells expressing CD107a, tumor necrosis factor, interferon-γ, and CD226.

## Discussion

PD-L1 is not expressed in most normal tissues, except for macrophages, however, its expression can be induced and maintained by many cytokines, especially interferon-γ^30^. High levels of expression of PD-L1 was reported in many human tumors^30^, and during an active antitumor immune response, PD-L1 could provide a mechanism of tumor escape^31^. PD-L1 expression is an indicator of poor prognosis for patient survival^32^, moreover, the response of anti-PD-L1 mAb was more correlated with PD-L1 expression on immune cells such as macrophages, dendritic cells, and T cells, other than tumor cells^33^.

Avelumab, a human IgG1 anti-PD-L1 mAb was developed and approved by FDA in 2017 for treating Merkel-cell carcinoma. Clinical analysis revealed that Avelumab could lyse human lung tumor cell lines, such as H460, and H441, but not cancer patient autologous PD-L1-positive PBMC, even the expression level of PD-L1 was similar to that of H460^28^. It seems that anti-PD-L1 mAb with competent Fc can function both as checkpoint blockade and tumor cell-specific killer. In addition, accounting to the official website of CStone Pharmaceuticals, they retained the ADCP of an approved anti-PD-L1 antibody (Sugemalimab), to induce direct tumor killing by macrophages and enhance tumor antigen presentation for long-term anti-tumor immunity.

In the clinical trials most anti-TIGIT mAbs used IgG1-Fc, such as Tiragolumab (Roche), Vibostolimab (Merck), and Ociperlimab (BeiGene)^34^, a common mechanism was the selective depletion of TIGIT-overexpressing Tregs within the tumor microenvironment^20^. Beyond ADCC/ADCP of IgG1-Fc, researchers found that cytotoxic T-lymphocyte-associated protein 4 (CTLA-4) and TIGIT mAbs required human CD16a engagement on APCs for optimal T cell activity. In vitro human PBMC stimulation revealed that IL-2 secretion was dependent on CD16a-hIgG1 interaction, and DLE-mutant Fc even increased IL-2 production. Anti-TIGIT mAbs with functional Fc could help to form the immune synapse of T cells and APCs^21^.

One of the concerns in the development of immune checkpoint inhibitors is the cytotoxicity mediated by Fc, for the reason of lysis of PD-L1^+^ and TIGIT^+^ immune cells. However functional Fc also could provide additional MOA of anti-tumor activity.

Here, we developed a tetravalent bispecific antibody targeting PD-L1 and TIGIT, armed with a fully functional Fc. We took the advantage of VHH to select the best bispecific antibody format and linker with optimal developability and cytotoxicity. We found that the association of heavy chain (HC) and light chain (LC) was significantly impacted by the location of VHH, when the fusion site was on the light chain, no matter how long the linker was, 7–17% heavy-chain only product mixed in the purified BsAbs (Fig. 2I–Q). The VHH binding ability also suffered a loss in the flow-cytometry assay (data not shown). The constant region of LC separated variable region of the light chain (VL) and VHH, the longest linker (GGGGS)3 is about 5.7 nm^23^, so there was no reason for VHH to pair with VL in cis.

A few reports investigated the relationship between BsAb formats and ADCC^35^. Researchers once developed tetravalent IgG1 dual targeting IGF-1R-EGFR antibodies based on IgG and single-chain variable fragment (scFv), they found that scFv fusion to the N terminus of HC or LC led to a low yield of BsAbs, which was not observed in our BiPT-22–25 (Table 1). Moreover, the attachment of scFv to the C terminus of HC completely abolished the cytotoxicity of bispecific antibodies, although the affinity of scFv only dropped 2 times. In our HC-VHH orientation formats, the fusion of VHH to HC made no difference to the Fc-FcγR interaction (Fig. 3), and the ADCC activity was positively related to the binding ability of VHH (Fig. 4). It seems that the best fusion site for scFv was the C terminus of LC, for VHH, the N terminus of HC was the best choice in our work.

There are several solutions to stabilize VHH, including grafting complementarity-determining regions into stable frameworks, the introduction of non-canonical disulfide bonds, random mutagenesis followed stringent selection, and point mutation of negative or positive charges, and genetic fusions^36,37^. Researchers once used the acid tail of α-synuclein (ATS) as a fusion to single-domain antibody (sdAb) lacking disulfide bond, which helped to stabilize the sdAb^38^, it was believed that lowering the isoelectric point (pI) of sdAbs could increase their stability^39^. However, in our work, we found that the fusion of VHH to the HC of YN035 improved the binding performance of VHH after the heating challenge (Fig. 4), the same results also can be observed in the 40 °C long-term stability assay (data not shown). The pI of YN035 was calculated to be 8.54, VHH was 8.34, BiPTs was about 8.50, VH of YN035 was 9.20, and HC of YN035 was 8.77, variable region of the heavy chain (VH) plus VL was 8.61, there was no reason to lower the pI of VHH by IgG. On the contrary, other reports revealed that positively charged VHH may be more aggregation-resistant^37,40^, which was coincidental with our result.

In vivo, BiPT-23 significantly suppressed tumor growth, by enhancing the infiltration of CD8^+^ T cells, NK cells, and depleting TIGIT^+^ Treg cells. Interestingly, BiPT-23 did not show any decrease in CD11b^+^F4/80^+^ cells, including dendritic cells (DCs) and macrophages. PD-L1 expression by DCs is a key regulator of T-cell immunity in cancer, PD-1 signaling can restrict T-cell responses during the cross-presentation of tumor-antigens by DCs^41^. BiPT-23 may not only function as a blockade of PD-L1 and TIGIT but also strengthened the cross-talking between TIGIT-expressing T cells and PD-L1^+^FcRs^+^ DCs. To our knowledge, BiPT-23 was the first bispecific antibody optimized for ADCC activity and had a potential for clinical development.

In the future, our bispecific antibody may combine with cytokines enhancing NK proliferation and function such as interleukin-2 and interleukin-15 to potentiate NK-mediated ADCC against antibody-coated tumor cells and T regular cells.

## Methods

### Ethics statement

293 T cell line was purchased from American Type Culture Collection (ATCC).

The PBMCs were extracted from healthy donors, and the informed consent was obtained from them.

This study with mice was conducted by Biocytogen in accordance with AAALAC guidelines. This study was approved by Biocytogen’s Institutional Animal Care and Use Committee and in compliance with the Guide for the Care and Use of Laboratory Animals. At the end of the experiment, animals were euthanized with an excess of CO_2_.

### ARRIVE compliance

All studies in this paper were carried out in compliance with the ARRIVE guidelines.

### Expression and purification of bispecific antibodies

Supported growth of EXPI293 to the density of 1.6 × 10^6^ cells/ml with OPM-293 CD05 Medium (OPM, 12112701), SMM293-TII (SB, RZ14JL0801), and 8 mM Gluta MAX-1 (100x) (gibco, 2085465), incubated in a 37 °C, ≥ 80% relative humidity and 8% CO_2_, 120 rpm orbital shaker. The next day EXPI293 cells reached a density of 3 × 10^6^ viable cells/ml. Using OPTI-MEM (gibco 2120548) to dilute plasmid DNA and 1 mg/ml PEI (plasmid DNA:PEI = 1:2), Gently inverted the complex 2–3 times, incubated at room temperature for 5 min. Added the diluted PEI to diluted plasmid DNA, put the mixture at room temperature for 20 min, and then transferred the solution to cell culture. After 5-day incubation, centrifuged the culture at 8000*g* for 10 min, filtrated supernatant with a 0.5 μm filter (cobetter, OES0423).

Turned on AKTA avant 25(GE), connected 10 ml Mab SelctSure (GE) column to the system, set the flow rate at 3 ml/min, washed the column with 0.5 M NaOH, and 1×PBS one after another until baselines were flat, then ran sample through the column. When sample supernatant went through the column system, washed the column with 1×PBS until baselines were flat, used 0.1 M Gly-HCl, pH3.0 to elute protein, neutralized with 1 M Tris–HCl. Connected 50 ml G-25 desalting column to the system, set flow rate at 8 ml/min, and washed the column with 0.2 M NaOH and 1xPBS one after another until baselines were flat. Collected protein, filtrated the collection through a 0.22 μm filter (Pall, FG1375), determined concentration by BCA Protein Assay kit (Thermo, UJ292598).

### SEC

Turned on the high-performance liquid chromatography (Agilent Technologies 1200 Series), First used MilliQ pure water with a flow rate of 2 ml/min to remove the air in the system pipeline, connected the SET-C SEC-300 (Sepax, 2F36102) to the instrument after washing for 30 min. Then cleaned the column with MilliQ pure water at a flow rate of 0.5 ml/min for 1 h, and used PBS at a flow rate of 1 ml/min for another hour. Diluted antibodies to 1 mg/ml, take 100 μl of the marker and the diluted antibodies into the sample tube respectively. Set the marker loading volume to 6 μl, flow rate 1 ml/min, and ran for 20 min, then set the antibody loading volume to 10 μl, flow rate 1 ml/min, and ran for 20 min.

### Flow cytometry

After digestion and centrifugation of human TIGIT-expressing 293 T and human PD-L1-expressing 293 T cells, they were resuspended in PBS containing 0.1% BSA, and 4E4 cells were added to a 96-well plate, 30 μl per well. Diluted the corresponding antibodies to 200 nM, and then diluted it by 3 times in 11 gradients, with no antibody added to the last gradient. Mixed 30 μl of antibody solution with the cell suspension, and incubated at 4 °C for 1 h. Washed the cells twice with PBS containing 0.1% BSA, and centrifuged at 500*g* each time for 5 min. Diluted the anti-human IgG Fc-650 secondary antibody (abcam, catalog number: ab98593) with 0.1% BSA-containing PBS at a 1:200-fold dilution, added 30 μl to each well, and incubated at 4 °C for 30 min. Washed the cells 3 times with PBS containing 0.1% BSA. Finally resuspended the cells with 30 μl PBS containing 0.1% BSA, read the data by the iQue machine, and used Graphpad to make a four-parameter fitting curve to calculate EC50.

### Stability assays

Diluted BiPT-22, BiPT-23, BiPT-24, BiPT-25, YN035, and hTIGI7.11E into 100 nM with PBS respectively, then incubated at 20 °C, 30 °C, 40 °C, 50 °C, 60 °C, 70 °C, 80 °C, 90 °C for 1 h, did flow cytometry at the concentration of 25 nM to detect the binding ability of all antibodies.

### ForteBio octet

The dual-target binding of the BiPT-23 was measured using BLI on the OCTET RED384 instrument (Sartorius). 100 nM BiPT-23 was captured for 200 s by Anti-Human Fc Capture (AHC) biosensor (Sartorius AG). The binding concentrations of PD-L1 and TIGIT were 100 nM, with 400 s of association phase, and 800 s of dissociation phase, followed by 5 s of regeneration in Glycine solution (pH = 1.5).

### Cytotoxicity

293 T cell lines stably expressing both human TIGIT or PD-L1 and luciferase were constructed, and the target cells were counted, centrifuged, and resuspended in a "DMEM + 10% FBS" medium, 5000 cells per well of target cells. Rinsed PBMC 3 times with PBS, 500*g* centrifugation, for 5 min, 400*g* centrifugation, for 5 min, 300*g* centrifugation, for 5 min, all centrifugations were conducted at room temperature. The effector cells were resuspended in a "DMEM + 10% FBS" medium, 1.75E5 cells per well, and the ratio of effective and target cells was 35: 1. Diluted antibodies to the initial working concentration: 100 nM, eightfold dilution, 7 concentration gradients, 50 μl/well. Mix target cells, effector cells, and antibodies in a 96-well cell culture plate with opaque white background, a separate well of the target cell was set as a control at the same time. After 48 h incubation in a 37 °C incubator, the content of luciferase in the plate was detected with a Tecan reader. Lysis percentage (%) = (target cell well n-antibody well m)/target well n × 100.

### Animal studies

5 × 10^5^ MC38 cells resuspended in PBS were subcutaneously implanted into the right flank of female TIGIT-humanized C57BL/6 mice. When the mean tumor volume reached 100 ± 50 mm^3^, mice were selected based on tumor volume and body weight. Qualified mice were randomly assigned to 5 experimental groups, with 6 mice in each group. Mice were treated with indicated proteins or human IgG1-Fc at 10 mg/kg once every 3 days for a total of 6 times. Tumor volumes were collected using the Vernier caliper, and volumes were calculated by use of the modified ellipsoid formula ½m length n width. After experiment, the mouse tumor tissue was collected to prepare a single-cell suspension, which was stained with LD-eF506 and antibodies: anti-mCD16/32, anti-mCD45-APC/Cy7, anti-mCD3ε-PerCP/Cy5.5, anti-mCD4-PE, anti-mCD8a-BV605, anti-mNK1.1-BV421, anti-m/hCD11b-APC, anti-mF4/80-FITC, anti-m/rFoxp3-PE/Cy7. TGI_TV_(%) = [1 − (Ti − T0)/(Vi − V0)] × 100% (Ti: the mean tumor volume of the treatment group on the i day of administration, T0: the mean tumor volume of the treatment group on the 0th day of administration; Vi: the mean tumor volume of the IgG1-Fc control group on the i day of administration, V0: the mean tumor volume of IgG1-Fc control group on the 0th day of administration). Data were shown as Mean ± SEM and analyzed using one-way ANOVA followed the Tukey test compared with each group (*p < 0.05, ***p < 0.001).

## Data Availability

All data are contained within the article.

## Abbreviations

BsAbs: Bispecific antibodies
ORR: Overall response rate
OS: Overall survival
VHH: Variable domain of the heavy chain of heavy‐chain‐only antibody
PD-L1: Programmed death-ligand 1
TIGIT: T cell immunoreceptor with Ig and ITIM domains
mAb: Monoclonal antibody
CTLs: Cytotoxic T lymphocytes
NK: Natural killer
ADCC: Antibody-dependent cellular cytotoxicity
ADCP: Antibody-dependent cellular phagocytosis
HC: Heavy chain
LC: Light chain
APC: Antigen-presenting cell
DC: Dendritic cell
TME: Tumor microenvironment
TILs: Tumor-infiltrating lymphocytes
Tregs: Regulatory T cells
DLE: Ser239Asp/Ala330Leu/Ile332Glu of IgG1-Fc
EC50: Half maximal effective concentration
IgG: Immunoglobulin G
MOA: Mechanism of action
FDA: U.S. Food and Drug Administration
PBMC: Human peripheral blood mononuclear cell
PVR: Poliovirus receptor
CTLA-4: Cytotoxic T-lymphocyte-associated protein 4
pI: Isoelectric point
VH: Variable region of the heavy chain
VL: Variable region of the light chain
scFv: Single-chain variable fragment
DCs: Dendritic cells
